# Considerations on establishing prevention reporting at the national level in Germany

**DOI:** 10.17886/RKI-GBE-2017-087

**Published:** 2017-08-30

**Authors:** Ursula von Rueden, Kevin Dadaczynski

**Affiliations:** 1 Department 2-25: Research, Quality assurance, Federal Centre for Health Education, Cologne, Germany; 2 Department 5-53: Research and Quality assurance, Federal Centre for Health Education, Cologne, Germany

## Abstract

The adoption of the Preventive Health Care Act in July 2015 was a key step in strengthening settings-based health promotion and disease prevention. This increases the importance of developing prevention reporting at the national level. In light of international experiences, we therefore propose a multi-step process, which formulates specific goals based on epidemiologically grounded public health needs, which should then be addressed through appropriate intervention strategies. The implementation status of activities needs to be continuously documented and their effectiveness evaluated.

In Germany, numerous stakeholders and institutions at the federal, state and local levels implement health promotion and disease prevention measures. In accordance with the World Health Organization’s Health in All Policies [1] strategy, effective and long-term measures to promote population health can only be achieved through targeted efforts across all policy fields. Properly addressing this task for the whole of society will require improving key prerequisites, such as agreeing on binding definitions for goals, coordinating and linking measures, ensuring the complete documentation of interventions and their results as well as also accounting for quality standards.

The Federal Centre for Health Education (BZgA) was established based on a decree in 1967 with the mission to maintain and improve population health in Germany. The work of the BZgA can be divided into three main areas, which are closely linked and mutually dependant:

1. Communication: planning and implementation of national prevention campaigns and programmes as well as the implementation of national action plans and statutory tasks
2. Cooperation/coordination: cooperation with state and non-state health care institutions, research institutes and economic institutions at the national and international level across all sectors and levels
3. Quality assurance and research: contributions on the efficacy of disease prevention and health promotion through quality assurance and evaluation.

In the context of the Preventive Health Care Act, the BZgA has, since 2016, supported the work of Germany’s National Association of Statutory Health Insurance Funds (GKV-Spitzenverband) to fulfil its tasks regarding settings-based health promotion and disease prevention. GKV-Spitzenverband commissions the BZgA to develop and implement health promotion and disease prevention measures collaboratively funded by different health insurance groups. Evaluating the nature and quality of these measures is also part of the BZgA's mandate. A central goal of the law amendment remains to enhance target-oriented cooperation between stakeholders. To this end, the National Prevention Conference set out national framework recommendations for all funding institutions to implement effective and target-oriented health promotion and disease prevention measures.

The Prevention Status Reports by the US Centers for Disease Control and Prevention could serve as a starting point for the development of national prevention reporting [2]. These online reports document the implementation of evidence-based public health strategies regarding relevant health concerns (such as overweight/obesity, heart attacks and strokes and/or tobacco consumption) for each US federal state. The reports all follow a pre-defined structure. Based on the available epidemiological data, they begin by describing a health concern, then, based on national recommendations and research, identify potential solutions, and finally provide an assessment of the implementation status of these solutions using a traffic light system.

Applied to Germany, a four-tier system for national prevention reporting would be conceivable (Figure 1):

1. Concerns/prevention needs: The successive expansion of Federal Health Reporting over the course of the past decades provides substantial data for a reliable assessment of disease prevention and health promotion needs within a settings approach. This includes health and epidemiologic surveys (such as the Robert Koch Institute’s health monitoring surveys – the German Health Interview and Examination Survey for Children and Adolescents (KiGGS), the German Health Interview and Examination Survey for Adults (DEGS) and the German Health Update (GEDA) – or the BZgA’s drug affinity study), as well as data from registries (such as the cancer registry), official statistics (such as micro census data) or routine data [3].
2. Health goals: Based on the findings of federal health reporting and further surveys, national health goals are already being developed and constantly updated at the federal level (www.gesundheitsziele.de). Related strategies and measures (for example intermediary goals and initial measures within the framework of health target processes) aim to stimulate the implementation of activities to enable the achievement of the defined goals [4]. Alongside national health goals, and based on preventive health care legislation, we should also mention the federal framework recommendations adopted by the National Prevention Conference [5]. These define life course-oriented goals, priority fields of action and target groups, as well as the participating organisations and their individual reporting duties.
3. Interventions: Whereas earlier findings and preliminary work provided a basis for previous stages, no consensus has so far been established on which concrete evidence-based intervention strategies we should focus on (for example to promote child and adolescent mental health). Numerous intervention-based quality procedures and instruments exist [6], as well as recommendations for specific fields of action [7]. However, these can only provide rudimentary guidance. So far, there is little transfer of research findings into evidence-based practices and this remains a further challenge.
4. Status: For prevention reporting, consensually agreed evidence-based intervention strategies that focus on specific goals and the development of corresponding indicators are important prerequisites. The description of implementation status should document the implementation of measures and evaluate their impacts, and also include an assessment and comparison of the desired and current state. So far, the implementation of the first two steps has come up against very clear barriers. There is a lack of standardised procedures to document implemented disease prevention measures that would allow for sufficient detail. Moreover, no consensus on how to evaluate methods, outcomes and instruments, particularly of complex, settings-based disease prevention measures, has so far been achieved. This severely limits the comparability of results. In the sense of a cyclical approach, the comparison between the desired and current state provides the basis for an updated description of disease prevention requirements.

## Outlook

All ideas introduced in this paper should serve as food for thought regarding the establishment of prevention reporting at the national level. Against the backdrop of current international activities, a multi-step approach appears to make sense. However, such an approach needs to be adapted to the particularities of the national context. Finally, it is necessary to take into account that expanding national prevention reporting will require the target-oriented collaboration between all relevant stakeholders in interdisciplinary disease prevention and health promotion.

## Figures and Tables

**Figure 1:**
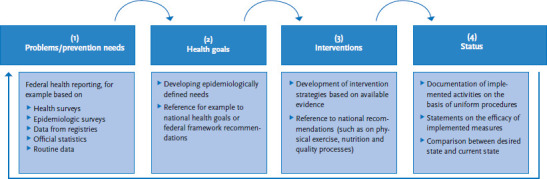
Multi-step implementation of national prevention reporting Own diagram
